# Photoinduced Processes as a Way to Sustainable Polymers and Innovation in Polymeric Materials

**DOI:** 10.3390/polym13142293

**Published:** 2021-07-13

**Authors:** Roberta Bongiovanni, Sara Dalle Vacche, Alessandra Vitale

**Affiliations:** 1 Department of Applied Science and Technology, Politecnico di Torino, 10129 Torino, Italy; sara.dallevacche@polito.it (S.D.V.); alessandra.vitale@polito.it (A.V.); 2 INSTM—Politecnico di Torino Research Unit, 50121 Firenze, Italy

**Keywords:** photopolymerization, photopolymers, photoinduced processes

## Abstract

Photoinduced processes have gained considerable attention in polymer science and have greatly implemented the technological developments of new products. Therefore, a large amount of research work is currently developed in this area: in this paper we illustrate the advantages of a chemistry driven by light, the present perspectives of the technology, and summarize some of our recent research works, honoring the memory of Prof. Aldo Priola who passed away in March 2021 and was one of the first scientists in Italy to contribute to the field.

## 1. Introduction

**Figure 1:**
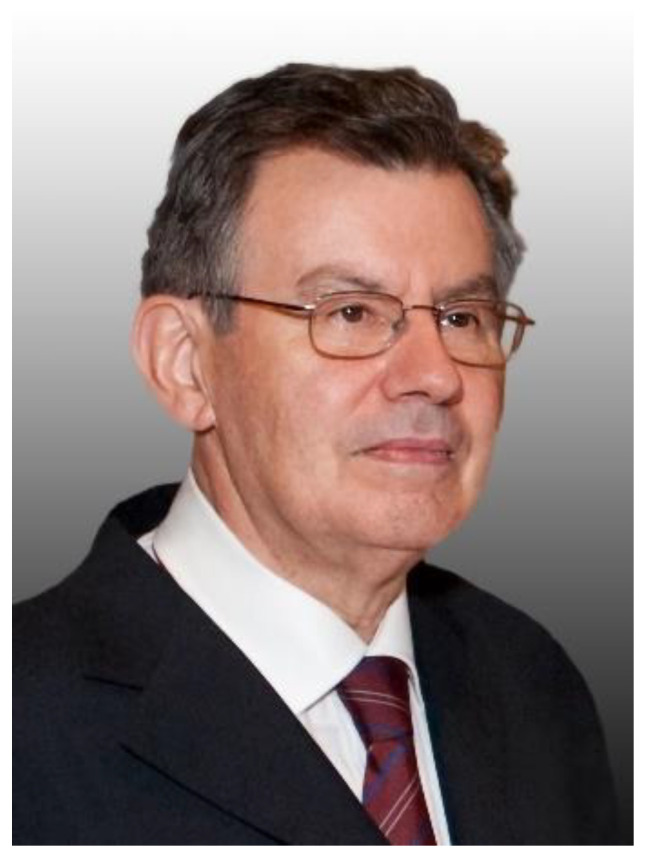
Photograph of deceased Prof. Priola.

This paper is in memory of Aldo Priola (Figure 1), full professor of chemistry at Politecnico di Torino, who passed away in March 2021. The research lines of our group, of which here is an account, are still inspired by his work on photopolymers and photoinduced polymerization.

Anyone who met Prof. Priola remembers him as a good man, very reserved and modest, despite his above average intelligence. He graduated in Industrial Chemistry at the University of Turin, and after a brief period at the CNR, he worked at the ENI research center in San Donato Milanese, leading a working group on cationic polymerization. His academic career began as Adjunct Professor at Politecnico di Torino: he was called on to give courses on polymeric materials and was subsequently appointed Associate Professor of Industrial Chemistry. After taking up a professorship in Messina, he returned to Turin as Full Professor of Chemistry. He led a group devoted to the photoinduced polymerization and characterization of polymers, choosing a new approach: when the science of materials was still in its infancy as an academic discipline in Italy, he understood the importance of studying the relationship between the engineering properties of materials and their chemistry. Sharing this idea with colleagues from industry and academia, he co-founded CISM (Consorzio Interuniversitario Scienza Macromolecolare), which later merged into INSTM; he was also among the founders of AIM (Associazione Italiana di Scienza e Tecnologia delle Macromolecole) and promoted the Materials Engineering Course at Politecnico di Torino. Deeply convinced of the importance of teaching and of the role of chemistry in the training of engineers, he contributed to the birth of AICIng (Associazione Italiana Chimica per l’Ingegneria). To him, we owe a lot of significant papers and many patents, but above all the merit of having taught the principles of honesty and integrity in all stages of scientific practice.

Prof. Priola published extensively in the field of photopolymerization. In this paper, we present photoinduced processes regarding polymers, and their potential applications, in particular, we highlight them through a series of examples from our recent work addressed to the preparation of biobased composites, advanced coatings and electrospun membranes.

## 2. The Birth of Photoprocesses

In his seminal work at the University of Bologna, Prof. Giacomo Ciamician (born in Trieste in 1857 and died in Bologna in 1922) conceptualized the development of a new chemistry based on light, able to revolutionize the world economy, by switching away from fossil fuel resources to renewable energy sources. His thoughts were discussed in a famous lecture at the International Congress of Applied Chemistry in New York in 1912, which made a lasting impression, was printed in several languages and was published in an issue of Science in the same year [1]. The paper is both visionary and prophetic: apart from the text style and the reference to a coal-based economy instead of an oil-based economy, the content seems to be taken by the present media, discussing sustainable development, circular economy and renewable processes. This extraordinary chemist anticipated several of the tenets of Green Chemistry: to plan reactions under mild conditions; to choose activation conditions for direct reactions assuring atom economy; to use renewable materials or reagents employing light; to reduce energy consumption. The similarity between Ciamician’s statements and the 12 principles of Green Chemistry, as formulated by Anastas and Warner in [2], are astonishing, in particular: (2) atom economy; (3) less hazardous chemical synthesis; (5) safer solvents and auxiliaries; (6) design for energy efficiency; (7) use of renewable feedstocks.

According to his vision, Ciamician developed over 30 years of pioneering work using light as a reactant in chemical processes: a very famous picture shows Ciamician making experiments on the sun deck of his department in Bologna to prepare organic compounds. In a series of papers on the chemical action of light [3,4,5,6,7], he clearly demonstrated that light has a main role in a chemical laboratory as it does in nature, namely in the leaves of plants. The reactions that Ciamician studied with his associate Paul Silber are hydrogen abstraction by carbonyls promoting reduction, reductive dimerization and reductive alkylation, and by nitro-compounds, α-cleavage of ketones, [2 + 2] cycloaddition of olefins, oxygenation and double-bond isomerization. From those times, many other photochemical methods have been developed further demonstrating that they are highly effective for the synthesis of organic compounds [8]. In fact, UV and visible photons have an energy content comparable to that of chemical bonds: upon absorption, electronically excited states are formed and start different chemical paths leading to chemical transformations of synthetic interest. The advantage of photochemical reactions over thermal reactions is that they have unparalleled mild conditions; first of all, no heating is required, moreover often aggressive chemicals, needed to promote the desired reaction, are avoided, further reducing waste. This means that the synthetic chemists have simpler work up and easier purification procedures; moreover, they have the chance to employ non-functionalized (or minimally functionalized) precursors, and to follow atom- and step-economical pathways. Ciamician himself remarked that «The photochemical reactions follow the fundamental laws of affinity, but have a special character. They are especially notable for the small temperature coefficient and are, however, comparable—a fact which is not without technical importance—to the reactions which take place at very high temperatures» [1].

## 3. Photoprocesses for Polymers

Since its birth as a discipline, photochemistry has consistently contributed to chemical synthesis, often complementing thermal processes. The advantages of photochemical reactions over thermal reactions have also been attractive for polymer chemistry, whose birth dates back to the 1920s: the landmark paper of Staudinger on polymerization was published in the same historical period of Ciamician [9]. Similar to Ciamician in the field of photochemistry, Staudinger, too, was visionary and able to see the potential of his ideas long before polymers and polymerization started to be fully exploited.

Light and polymer interactions are manifold. Looking at “IUPAC Glossary of terms used in photochemistry (IUPAC Recommendations 1996)” [10], one can find several terms referring to polymer synthesis, such as:

- photopolymerization: polymerization requiring a photon for the propagation step;
- photoinduced polymerization: polymerization of a monomer by a free radical or ionic chain reaction initiated by photoexcitation;
- photocrosslinking: photoinduced formation of a covalent linkage between two macromolecules or between two different parts of one macromolecule;
- photocuring: technical expression for the photoinduced hardening of a monomeric, oligomeric or polymeric substrate normally in the form of a film.

Other relevant terms related to the interactions between a polymer and light are photografting and photodegradation. Photografting is the modification of polymers by light-induced covalent bonding of monomers or polymeric side chains onto the backbone. i.e., a light-induced graft copolymerization [11]. Photodegradation refers to the photochemical degradation of a macromolecule into lower molecular weight fragments, usually in an oxidation process. Photodegradation implies end-of-life considerations and states a role of light in the recycling of polymers and in waste disposal.

The chemistry of photoinduced polymerization is described more in detail in the next paragraph.

### 3.1. Photoinduced Polymerization Chemistry

A complete overview of the photoinduced polymerization mechanisms is reported in [12]. Photoinduced polymerizations are mostly a type of chain-growth polymerization reactions, which include initiation, propagation and termination steps, where the generation of reactive species (initiation) occurs thanks to light. Usually, a photosensitive molecule is needed (photoinitiator), and in some cases, the monomer itself can initiate the reaction. The reactive species, either radical or ionic, react with monomers, oligomers and/or prepolymers: depending on their functionality, linear or crosslinked polymers are formed, i.e., if the reactive system is multifunctional, a curing reaction takes place (known as UV curing as most of the time UV light is used).

**Scheme 1:**
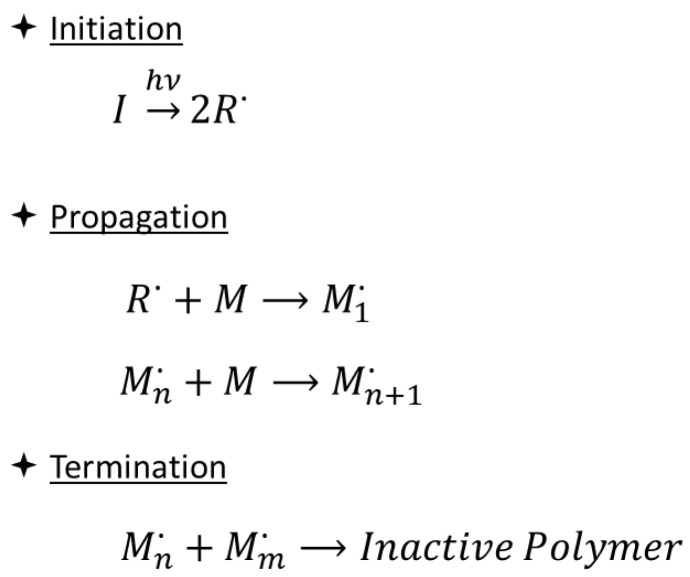
Mechanism of free radical photoinduced polymerization reactions.

Currently, photoinduced polymerization processes most commonly involve a free radical reaction mechanism (Scheme 1), suitable for monomers with carbon–carbon double bonds, such as unsaturated polyesters or (meth)acrylic monomers [13]. In this case, the initiation step consists of the generation of radicals from Norrish Type I (homolytic cleavage) or Type II (hydrogen abstraction) photoinitiators upon irradiation with light of a suitable wavelength.

**Scheme 2:**
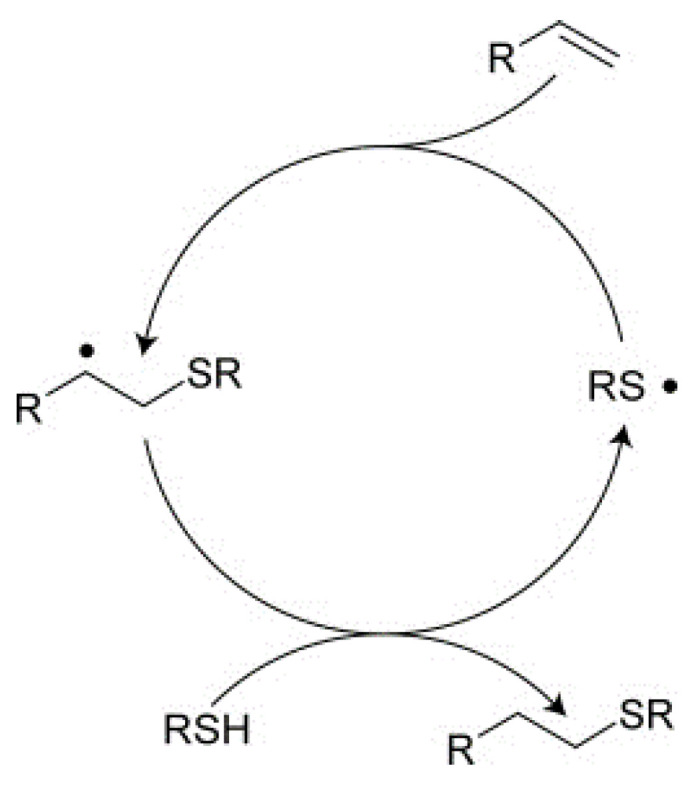
Mechanism of thiol-ene radical polymerization reactions.

This traditional photopolymerization process has broad applicability, but several drawbacks, namely oxygen inhibition, presence of unreacted monomer, polymerization induced shrinkage and subsequent stress development. These issues are overcome in thiol-ene-type photopolymerization reactions. The thiol-ene reaction proceeds, even without an initiator, alternating propagation and chain transfer reactions leading to a step-growth evolution of a network (Scheme 2).

Investigating the chemistry of difunctional acrylic model systems, Prof. Priola explored their structure-properties relationships when only some studies on pure well characterized diacrylate oligomers or dimethacrylate derivatives (from polyalkylenoxides) were available. He showed how by selecting the type of oligomer and its MW, it is possible to change the characteristics of polarity and flexibility of the polymeric networks obtained in the form of films, making them suitable for very different applications [14,15].

**Scheme 3:**
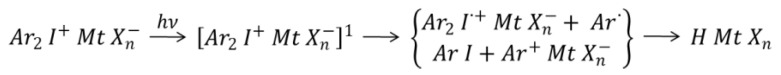
General mechanism for the photolysis of onium salts.

Having worked extensively on the cationic polymerization of rubbers as an industrial researcher at ENI (the Italian oil company), Prof. Priola investigated also the reactivity of several systems that do not polymerize through radical mechanism but are reactive in the presence of protons generated by light, such as epoxy, oxetanes and vinyl ethers [16,17,18,19,20,21,22,23,24,25]. In fact in the 1970′s, Crivello and coworkers first described the use of iodonium and sulfonium salts (onium salts) as initiators for photoinduced cationic polymerization reactions [26]. The photoinduced decomposition of onium salts (Scheme 3), followed by hydrogen transfer, results in the formation of Brønsted acids that are the actual initiating species in the polymerization reaction [27].

**Scheme 4:**
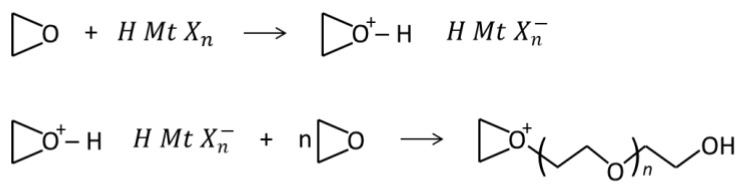
Mechanism of the cationic ring-opening polymerization of epoxide monomers.

The reaction then proceeds by a chain-growth mechanism, which depends on the type of monomer used. The most commercially relevant monomers for cationic photopolymerization are epoxides, which in the presence of a photogenerated acid undergo a ring-opening polymerization (Scheme 4).

Ionic photopolymerization is classified as a non-terminating reaction; with the correct process design, termination rates are low and the propagating species do not react with each other. The reaction thus may continue for long times after illumination is ceased (dark curing) [28]. This may be particularly useful for applications in which thick, colored or filled polymer systems are used, as reported by Prof. Priola in a paper that is one of the first examples of the use of frontal polymerization of epoxides [29].

Although there is potentially a wide range of industrially relevant base-catalyzed reactions, which have also the advantage of not being sensitive to oxygen and moisture, anionic photopolymerization is at the moment the less exploited mechanism. This is mostly due to the lack of suitable photoinitiator systems, although recently new concepts for photolatent bases have been reported [28,30].

In the past ten years, photoprocesses have also merged with new techniques resulting in the precise synthesis of complex polymer architectures. In fact, as discussed in the essay “The Next 100 Years of Polymer Science” [31], where the editors and advisory board of Macromolecular Chemistry and Physics reflect on the future of polymer science, an essential challenge for synthetic polymer chemists is finding new synthetic methods, namely precision synthesis, orthogonal chemistry, together with new polymerization methodologies, kinetics and digital materials design. They can certainly take advantage of light and the present literature already collects many works on original photomediated polymerization reactions [32]: photomediated atom transfer radical polymerization (ATRP), photomediated reversible addition-fragmentation chain transfer (RAFT), photomediated ring opening metathesis polymerization (ROMP), photoinduced nitroxide mediated radical polymerization (NMRP, also referred as stable free radical polymerization SFRP), photomediated polymerization–induced self assembly (PISA), photoinduced frontal polymerization (FP). In fact, as already mentioned, photomediation is more effective than thermal processes and light can drive precision macromolecular and materials chemistry. An excellent example is the preparation of single chain nanostructures by flow photochemical processes based on the photodimerization of anthracene units in polymethylmethacrylate under UV irradiation at 366 nm [33].

### 3.2. Advantages of Photoinduced Polymerization

Polymerization takes advantage of the specific benefits of light as the energy source to activate chemical processes, in particular temperature independence, extrinsic control through selection of the light source, temporal and spatial control. Indeed, photoinduction is more effective than thermal induction. As a rule of thumb, given a certain operational temperature, by using light, the energy available for the activation of the process can be orders of magnitude higher than in thermal processes. More important in view of demanding syntheses for precise and complex structures is the possibility to use different lights and having sources assuring high precision in wavelength and intensity, thus increasing control of the process and selectivity.

The availability of energy in mild conditions and the possibility of selecting the energy level turn into many advantages. First of all, the operations can be performed even at room temperature and this is obviously extremely favorable. Moreover, there are cases where a low temperature polymerization is essential. This constraint applies when the polymerization must take place on, or in contact with, thermosensitive substrates, e.g., the curing of a polymer coating on a plastic substrate, the fabrication of an implant in vivo, or the encapsulation of biological species and living cells inside a polymer. Another case requiring low temperature process is the synthesis of polymers using monomers with a low crystallization temperature. In all these situations, a photoinduced process is the only strategy to adopt.

Although room temperature is usually employed, photoinduced polymerizations are fast reactions so that even a few seconds of irradiation are enough for attaining quantitative conversions. Interestingly, rates can be extrinsically controlled through the change of light intensity and/or the switch to a different light wavelength.

The use of a light source instead of a thermal source also means space saving, as irradiation units are much smaller and more compact compared to ovens: this has a practical application when operating at a large scale and especially at an industrial level.

Another interesting aspect of photoprocesses is that they allow a temporal control of the reaction: while thermal processes due to heat and mass transfer reasons cannot instantaneously be switched on and off, photochemical processes are stop and go reactions, as light (i.e., one of the reactants) can be easily turned on/off. As a consequence, specific polymerizations can be activated on demand and transformations are more precise and efficient. The light source is extrinsic to the photochemical system, therefore the chemist can adjust both wavelength and intensity without changing other reaction parameters; this gives additional opportunities to activate and control the reaction. The development of novel and efficient light sources (e.g., LEDs), as well as photoinitiating systems (e.g., suitable for visible light irradiation) is continuously advancing [34,35]. Moreover, the opportunity to use different wavelengths has also been explored: this approach permits the performance of chromic orthogonal reactions [36].

Furthermore, in a photoinduced polymerization, the energy available is taken by specific light harvesting species, unlike thermal energy which is transferred through the entire reaction mixture of monomers and oligomers. This means that the reactions are also spatially controlled: while being very rapid, they take place only in areas where there is irradiation. The remarkable consequence of this feature is that photoinduced polymerization reactions are suitable for patterning and additive manufacturing processes. In fact, they are adopted in techniques such as stereolithography (SLA), digital light processing (DLP), continuous liquid interface production (CLIP), direct laser writing (DLW): they are effective tools for the printing of precise and complex 3D architectures at the micro and nanometer scale but are also the gateway to 4D printing, which is the 3D printing of adaptive and dynamic structures and is considered a great challenge in today materials science [37]. As the additional dimension means the ability of the printed structures to change shape and/or properties in a controlled fashion when subjected to an external stimulus, light again is a fascinating and leading method [38].

## 4. Shaping Polymers with Light

**Figure 2:**
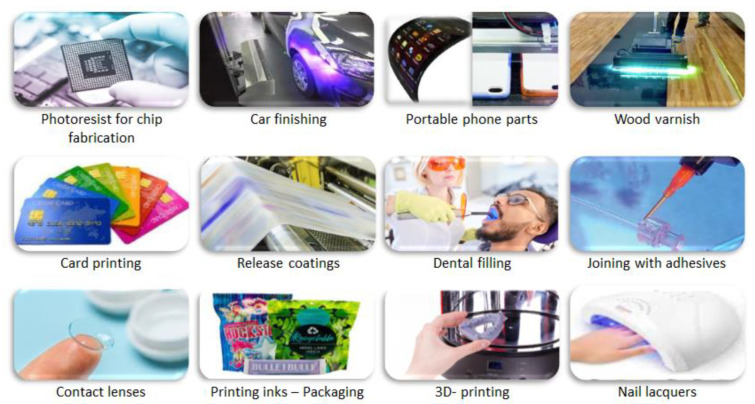
Examples of applications of photopolymers.

In general, photoprocesses, and especially photocuring processes, are essential in ordinary industrial production, being often the most competitive tools for making materials. They have many long-established applications and are therefore commonly used in everyday life and ubiquitous, as shown in Figure 2.

Due to the intimate control in both time and space, photoreactions can be coupled with patterning processes and 3D printing. Our group demonstrated in several works that photoinduced processes are suitable to develop innovative patterning techniques and that photopolymers are the materials of choice for the application of patterned structures in different fields, such as advanced coatings and microfluidics [39,40,41].

**Figure 3:**
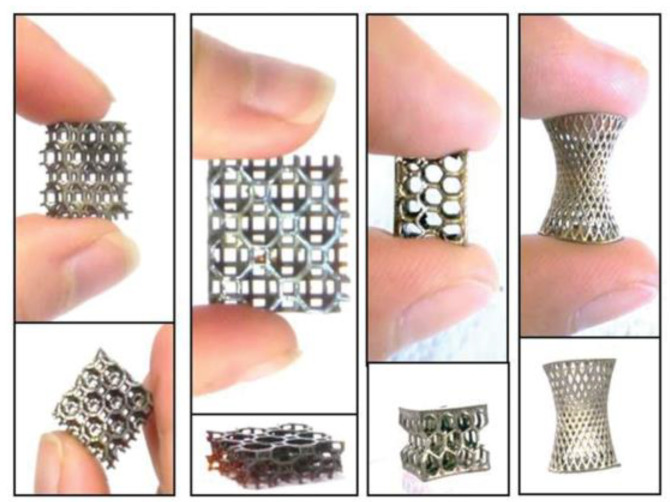
Different types of Ag NPs–polymer composite 3D printed structures. Reprinted from [42].

As an example of devices obtained by shaping polymers with light, in Figure 3 the outcome of our collaborative work is reported: a conductive 3D device produced by photopolymerization-based additive manufacturing [42]. The device is fabricated layer-by-layer by a DLP system, where the transformation of the photocurable liquid oligomers into a 3D object is based on their spatially controlled solidification induced by light. A low cost commercial DLP printer was used without any modification: after the optimization of the photocurable formulations, making them suitable to be processed by the printer, complex micrometer-scale electromechanical objects, guaranteeing the requested performance, were obtained. In particular, in the work, a metal precursor was dissolved in a liquid acrylic oligomer containing a dye and a two-components radical photogenerating system. The first component, working in the near UV-violet-blue spectrum (i.e., centered at 405 nm), initiated the photopolymerization as the DLP system worked with an illumination source in the visible range. At this stage, the role of the dye was to prevent the leaking out of light from the desired illumination area; moreover, it allowed to control the thickness of each layer during the printing process (i.e., z-resolution) by tuning the penetration of light into the resin and thereby the curing depth. The second photoinitiator, with absorption in the deeper UV spectrum, was activated when the printed object was subjected to a post-curing step with a 365 nm light. The newly generated radicals, in addition to further enhance the polymer conversion, supported the reduction of metal ions to metal nanoparticles: the in situ nucleation of the nanoparticles happened by electron transfer from the free radical to the metal cations. At this stage, the dye under UV irradiation decolorized without hindering the photoreduction of the metal ions. Therefore, during the light exposure conductive nanoparticles were generated in situ by photoreduction of metal precursors, while directly embedded in a polymeric matrix formed by photocrosslinking the acrylate oligomers during the printing stage [42].

Hence, it was demonstrated that coupling photoprocesses with the 3D printing technology opens several possibilities in a broad range of fields and enables the design and creation of geometrically complex objects with tailored topology, and thus functionality. The method can complement other techniques already proposed in the field of 2D or 3D printing of conductive composite materials: it has the advantage of being a low cost, fast and versatile manufacturing process suitable for the production of dissipative structures and packaging materials, which can be integrated into electronic devices or envisaged for similar applications.

**Figure 4:**
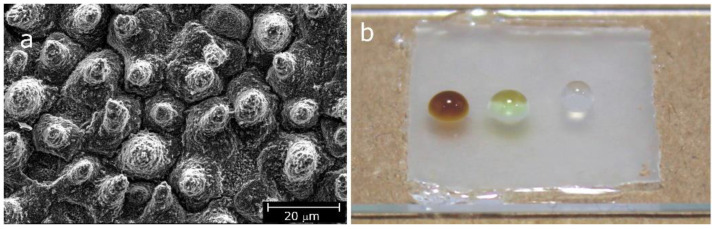
(**a**) Scanning electron microscope image of a synthetic replica of a lotus leaf produced by UVNIL with an acrylate monomer with 5 wt.% of a polyether siloxane urethane diacrylate comonomer, applying a creep at 3 bar for 5 min. (**b**) Coffee, olive oil and water droplets (from left to right) on a siloxane synthetic lotus surface. Reprinted with modifications from [43].

Patterning techniques based on photopolymerization can also be used to prepare functional polymer surfaces, which can be applied in the field of coatings. An example is reported in another collaborative work [43] of our group, where a hierarchical patterned polymer surface was fabricated by light-induced nanoimprinting (Figure 4). The pattern of Figure 4 is a bio-inspired fluorine-free and self-cleaning polymer coating, developed using a combination of self-assembly and low-pressure UV nanoimprint lithography (UVNIL) process. Adding an acrylic siloxane to a UV curable acrylate oligomer, a spontaneous migration of the low surface energy comonomer towards the polymer–air interface enabled to create hydrophobic surfaces with a water contact angle of 105°. Against this surface, templates, which were prepared from superhydrophobic lotus leaves, were applied at low pressure. The lotus leaf hierarchical texture, comprising micropapillae and sub-micron crystalloids, was well reproduced in the copolymer material (Figure 4a). The synthetic replica with the texturized surface showed a water contact angle of 144°, which could increase to 152° with increasing creep time under pressure to 5 min prior to polymerization. As shown in Figure 4b, the synthetic lotus coatings demonstrated also good anti-stain properties. Moreover, in spite of a water sliding angle above 10°, the surface was self-cleaning: when contaminated with hydrophobic pepper particles, water droplets could remove them quite efficiently. The process and the material adopted in the work represent an efficient alternative to most self-cleaning polymers developed: in fact, neither fluorinated compounds nor nanoparticles, which create concerns for their biopersistency and for uncontrolled release issues, respectively, were used. Moreover, the process can be easily scalable to cost-effective, large area surfaces [43].

**Figure 5:**
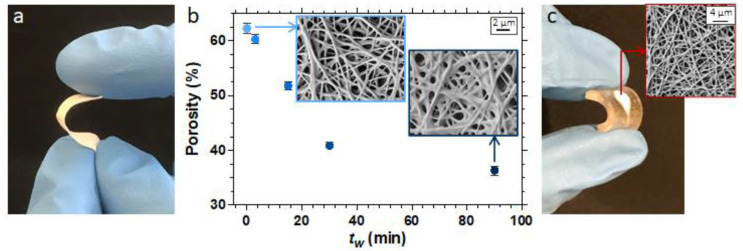
Photocrosslinked electrospun membranes made of polybutadiene nanofibers. (**a**) Picture of a fibrous membrane showing its flexibility. (**b**) Effect of the waiting time between the electrospinning step and the light exposure (*t_w_*) on the mat porosity; the insets represent FESEM images. (**c**) PDMS microfluidic device with an embedded electrospun functional membrane.

In our previous works, we also showed how photoinduced processes can be used in combination with electrospinning (i.e., a versatile technique to produce fine submicrometric fibers and nonwoven fibrous mats from polymer solution or melt through the application of strong electrostatic forces) [44,45,46]. Photo-crosslinking has been applied to modify various polymeric electrospun fibers, both by irradiating the fibers in situ during their fabrication and by irradiating the formed fibrous mat after electrospinning. The photo-crosslinking reaction is conducted to modify the physico-chemical properties of the fine fibers, e.g., preventing dissolution in water or other solvents, and to enhance their mechanical strength, as expected by any curing processes. Recently, we carried out a work on solution electrospinning of polybutadiene and irradiation of the rubber nanofibrous membrane to stabilize the fiber morphology [45]. In fact, as the *T_g_* of the polymer is low (*T_g_* ≈ −70 °C), there is a spontaneous cold flow that modifies the size and shape of the electrospun fibers. However, this problem was solved by photo-crosslinking the polymer fibers exploiting the thiol-ene chemistry, thus obtaining shape-stabilized rubber nanofibrous membranes (Figure 5a).

Moreover, it was possible to fine-tune the morphology of polybutadiene-based nanofibers, in terms of fiber size, membrane surface area, and its porosity (Figure 5b) by carefully selecting the electrospinning and photocuring process parameters (e.g., the flow rate, the time between electrospinning and irradiation, and the light dose). Even more interestingly, varying the composition of the electrospinnable solution, e.g., the type of polybutadiene, its vinyl content, the amount of thiol crosslinker, and the thiol/ene ratio, the fibrous membranes showed a different amount of active functional groups (i.e., double bonds or thiol groups), available both in the bulk and on the surface of the crosslinked nanofibers. Therefore, the rubber nanofibers turned to be active and functional nanomaterials suitable for further selective modification. Tuning functionalities to polymeric nanofibers in a controlled way is a promising topic for the technological development of electrospinning: evidently, the proposed method is simple and can be applied to other electrospinnable polymers and other functionalities, also changing the chemistry involved (e.g., azide−alkyne and Diels−Alder cycloaddition could be employed) leading to interesting applications. In our case, rubbery electrospun membranes were embedded into a polydimethylsiloxane (PDMS) microfluidic chip to fabricate a nanostructured functional device for (bio)sensing, as shown in Figure 5c [45].

## 5. Limitations of Photoprocesses and Potential Opportunities of New Developments

Photoprocesses in polymer chemistry have also limitations. The first one is that there is a short penetration depth and length dependency of light absorption. Therefore, thick samples are not easily processed, and the obtaining of 3D objects is done layer-by-layer. The spatial control, which is essential in 3D printing, means also that in many applications where dark areas are present (e.g., the crosslinking of adhesive or the curing of a varnish on a substrate with shadowed areas) polymerization remains uncomplete. Hence, different recent photopolymerization techniques have been developed to solve these issues of light penetration [27,47,48],

Moreover, the reactions depend on extrinsic parameters, sometimes leading to a lack of reproducibility unless the researchers are very accurate in reporting their results and the methodologies through which they were obtained. As a matter of fact, it is remarked that often the extrinsic parameters governing the polymerization process (e.g., light intensity and wavelength) are often omitted/forgotten by the researchers in their papers.

An important limitation of photoinduced polymerizations comes from the fact that most of them follow a radical mechanism, which is inhibited by oxygen [49]. This means that they are sensitive to air and have the expected efficiency only when performed in a controlled atmosphere [50]. Another drawback connected to the mechanism of polymerization, in particular the initiation step that relies on photoinitiators, is that their reaction can lead to species imparting a coloring to the final materials. Moreover, they can also form dangerous migrating byproducts. When photoinduced polymerizations imply ionic mechanisms, some of the above limitations are overcome, but rates are usually lower; more relevant constraint in adopting ionic photoinduced reactions for the synthesis of polymers is the availability of both monomers, oligomers and photoinitiators at a large scale.

Photopolymers can also suffer from limited mechanical properties; reinforcing fillers can thus be used [51], but their type and content have to be controlled based on the light absorption of the system [52].

Finally, photopolymers and photoinduced processes still pose sustainability issues, also in view of the rising tide of regulations in health, safety, and environment. In few words, current photoprocesses mostly rely on monomers, oligomers, photoinitiators coming from fossil resources, and many of the above chemicals are not safe: acrylics for radical processes are irritant; photoinitiators decompose into species which can uncontrollably migrate and be harmful [53,54]; most products are water insoluble, and when a dilution for rheology control is needed, it cannot be done with water (and the use of solvents gives rise to the VOC issue); the main light source employed for irradiation is UV, which is not safe both to the workers as it is harmful to the eyes, and to the environment as it develops ozone; most photopolymers currently produced are covalently crosslinked networks (i.e., they are irreversibly cured and hardly recyclable), thus non compliant with the present societal challenge of a circular economy.

It is evident that, in view of sustainable development, also photoinduced processes in the polymer field should be redesigned by (i) using biobased reagents and obtaining biopolymers, (ii) avoiding dangerous reactants (including dangerous light sources), (iii) assuring recyclability and/or reprocessability of the materials. Last but not least, the field needs the development of a comprehensive and general modeling framework and predictive models to describe and control photoinduced processes, especially for material manufacturing and patterning.

The points raised are nowadays clearly guidelines for research in the field. Regarding the use of innovative materials, several groups have undertaken works on biobased oligomers. We have recently synthesized and characterized a platform of methacrylic oligomers based on eugenol [55]: the reaction of the double bonds was affected by the presence and reactivity of the allyl and propenyl groups in the eugenol- and isoeugenol-derived methacrylates, respectively. It was proved that these groups are involved in radical addition, degradative chain transfer, and termination reactions, yielding crosslinked polymers.

**Figure 6:**
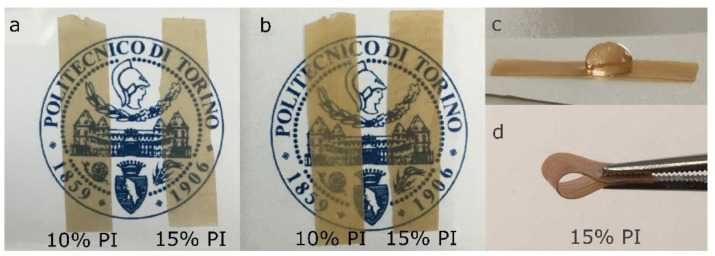
Composite of epoxidized cardanol and hemp nanocellulose with 10 wt.% and 15 wt.% photoinitiator (PI): (**a**) before curing and (**b**) after curing for 5 min of on each side; (**c**) water drop on composite with 15 wt.% PI cured for 5 min of on each side, and (**d**) a photo showing the flexibility of the cured composite. Reprinted from [57].

We have also developed composites made of epoxidized cardanol with nanocellulose (Figure 6): after irradiation, transparent, rubbery, flexible, and water repellent films with potential application in the packaging sector were obtained [56,57]. We discovered that a fairly high concentration of photoinitiator, of as much as 15 wt.%, was needed for leading the crosslinking reaction to completion, due to a side reaction of the acid species produced by the photoinitiator with the microfibrillated cellulose. Currently, cellulose-based composites with a polymeric matrix made of a biobased acrylic latex with coumarin units are also studied. The coumarin units allow a [2 + 2] cycloaddition, induced by irradiation with a specific wavelength. By photocycloaddition (i.e., the formation of dimers), crosslinking of the matrix thus insolubility is assured. Interestingly, it also gives a decrease of permeability to oxygen, which is an important property in view of using the material for packaging applications. At present, we are implementing the reversibility of the crosslinking by irradiation in view of dismantling the network and recycling the composite.

Moreover, the limitations and problems inherent to photopolymerization can be turned into opportunities. In the following, we report a couple of our works regarding the exploitation of oxygen inhibition and of the irradiation gradient to prepare peculiar materials.

**Figure 7:**
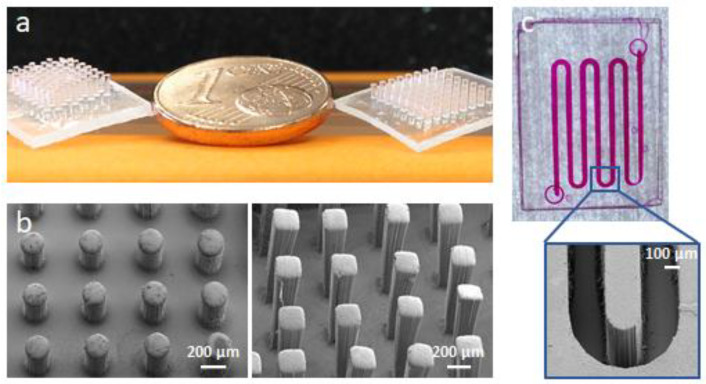
(**a**) Examples of mono- and multimaterial thick polymeric structures obtained through the OIL technology. The structures on the left are made of poly(ethylene) glycol diacrylate (PEGDA) and are circular pillars with a diameter of 200 μm and a height of 1.5 mm. The structures on the right have a 0.8 mm thick PEGDA bulk and are made of a perfluoropolyether dimethacrylate (PFPEDMA): the squared pillars are 1.2 mm high and 200 μm wide. (**b**) FESEM images of the thick patterns obtained by OIL. (**c**) Example of a PFPEDMA microfluidic device fabricated by OIL technology, filled with colored water-based solution, and a FESEM enlargement showing the curve of the microchannel.

Oxygen inhibition implies that during irradiation of oligomers undergoing polymerization by a radical mechanism, in the outermost layer (i.e., in contact with air) the reaction does not proceed and conversion is very low. Therefore, while the bulk becomes solid, the surface remains liquid and tacky, which is a serious drawback in any technological application, in particular coatings. Many solutions have been proposed to prevent the reactions of radicals with oxygen and the competition with the propagation reactions. However, the formation of the inhibition layer can be a tool to control the polymer surface. For instance, we coupled the formation of the inhibition layer with a photolithographic approach to generate polymeric patterns [58]. First, the areas to be UV irradiated are selected and consequently converted into solid polymer; as the unreacted inhibition layer has still reactive groups, it can be further processed through a sequence of irradiation steps in air with photomasks, selected depending on the geometry to be obtained, and a final step of exposure under inert atmosphere (e.g., nitrogen) assuring the complete curing of the surface layer. We called this method Oxygen Inhibition Lithography, and the only constraint is the use of monomers undergoing radical polymerization. In this way various patterned structures, even with high thicknesses, can be created (Figure 7): in fact, fresh monomer can be added on top of the thin undercured layer, and in this way the height of patterns depends only on the amount of liquid resin that is added for the construction of each layer. The interlayer adhesion is very good as the inhibition layer acts as an adhesion promoter, and during the irradiation step after the addition of the monomer, strong chemical bonds are generated between two adjacent layers. Even more interestingly, as shown in Figure 7, one can easily generate multimaterial structures, where each layer contains a different chemistry (as said before, monomers suitable for radical mechanism of polymerization need to be used). This means combining in the same device different sets of properties belonging to different polymers, ensuring an excellent adhesion between the layers although they are not made of the same material. The OIL technology is extremely versatile and simple. Versatility means that multiscale (from micro- to millimeter sizes) open-faced-patterned structures, and even closed devices (Figure 7c), can be obtained. Simplicity means that the process itself and the types of equipment required are simple and low cost, while both reproducibility and productivity are high [58].

**Figure 8:**
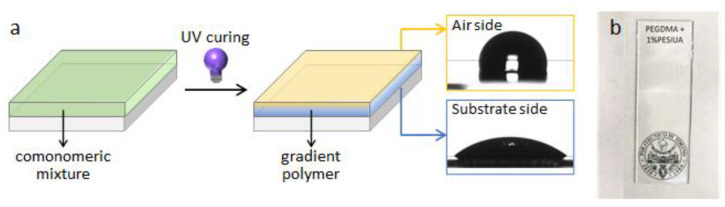
(**a**) Scheme of the photopolymerization process to obtain siloxane copolymers with compositional gradients by photocontrolled surface segregation, showing hydrophobicity on the air side, due to the low polarity siloxane comonomer, and hydrophilicity on the substrate side, due to the (meth)acrylic comonomer. (**b**) Photograph of transparent photocured poly(ethylene) glycol dimethacrylate (PEGDMA) with 1 wt.% polyether siloxane urethane diacrylate (PESiUA) film on a glass substrate.

As mentioned before, photopolymerization processes rely on irradiation of monomers and therefore are also governed by light intensity. Inside the reactive system along its thickness, light intensity shows a net gradient. In other words, intensity changes depending on the distance of the monomer from the light source. In turn, the conversion is also affected by a gradient and the properties of the polymer obtained, too. When monomers of different reactivity are copolymerized, interesting copolymers with a gradient composition are obtained. In fact, the monomer with the higher reactivity polymerizes faster at the surface of the film, where the light intensity is higher; as a consequence, it is depleted at the surface, and due to the difference in chemical potential it diffuses from the bulk to replenish the surface. At the same time, the lower reactivity monomer experiences a counterdiffusion from the surface into the bulk. This phenomenon, called photoenforced polymerization, results in an enrichment of the polymer surface with the monomer that reacts faster [59]. We applied this idea to comonomers having different wettability due to different polarities. Prof. Priola and his group for many years studied the surface of photopolymers and he was one of the first authors to selectively modify the surface by using fluorinated comonomers and characterize them by XPS techniques, demonstrating that there is a spontaneous migration of low surface energy monomers to the free surface [60]. Building on this concept of surface segregation, in combination with the photoenforced polymerization, we developed photocured gradient coatings with tunable surface chemistry and properties (Figure 8) [61]. We could independently and simultaneously control and modulate the surface and bulk properties of polymers, enabling the production of gradient materials that can be tailored for specific applications. The study was developed investigating the photocopolymerization of acrylates and methacrylates having either hydrogenated (high surface energy) or siloxane (low surface energy) chains. The surface enriching of the siloxane segments driven by thermodynamic forces pushing the apolar monomer to the free surface was optimized when copolymerization took place between the siloxane acrylate (reacting faster) and a hydrogenated methacrylic comonomer (reacting slower). The copolymers showed a surface tension as low as γ = 22 mN m^−1^, in the presence of 1 wt.% of siloxane comonomer (Figure 8). The surface was tuned by changing the light intensity and the light gradient in the film by modifying the film thickness: depending on the local polymerization kinetics, different surface compositions and surface wettabilities were obtained. For the best effectiveness of the low surface tension siloxane acrylic oligomer, copolymerized with methacrylic monomers, low light intensity and the presence of a substantial light gradient was necessary [61].

## 6. Concluding Remarks

Photomediated processes have been demonstrated to be relevant in polymer science. Specific architectures and specific properties can be obtained not only by synthesizing and selecting new monomers, but also by leveraging on the special features of light-activated processes. A large amount of research work in material science is therefore developed using light, also our research work has contributed to the preparation of new materials and devices by photoinduced polymerization processes.

The photochemical control of polymeric material preparation means challenges, but also opportunities: thus, photochemical approaches can be a relevant option for polymer chemists and material scientists for implementing the development of polymers and polymeric materials and their processing. In fact, thanks to its intrinsic features, in particular fastness and versatility, light driven polymerization becomes a key enabling technology within the 4th Industrial Revolution (“Industry 4.0”). It already contributes to a paradigm change in design, fabrication and distribution of goods (at least those produced in low volume) and can make manufacturing processes flexible enough to allow mass customization, i.e., to produce products according to customer needs while keeping acceptable costs. Finally, having the potential for being a green chemistry and green technology, photoinduced processes can highly contribute to sustainable development.

## References

[1] Ciamician G. (1912). The Photochemistry of the Future. Science.

[2] Anastas P.T., Warner J.C. (1998). Green Chemistry: Theory and Practice.

[3] Ciamician G., Silber P. (1901). Chemische Lichtwirkungen. Eur. J. Inorg. Chem..

[4] Ciamician G., Silber P. (1902). Chemische Lichtwirkungen. Eur. J. Inorg. Chem..

[5] Ciamician G., Silber P. (1907). Chemische Lichtwirkungen. Eur. J. Inorg. Chem..

[6] Ciamician G., Silber P. (1908). Chemische Lichtwirkungen. Eur. J. Inorg. Chem..

[7] Ciamician G., Silber P. (1908). Chemische Lichtwirkungen. Eur. J. Inorg. Chem..

[8] Lazzaroni S., Ravelli D., Protti S., Fagnoni M., Albini A. (2017). Photochemical synthesis: Using light to build C–C bonds under mild conditions. Comptes Rendus Chim..

[9] Staudinger H. (1920). Über Polymerisation. Berichte der Dtsch. Chem. Ges. A B Ser..

[10] Verhoeven J.W. (1996). Glossary of terms used in photochemistry (IUPAC Recommendations 1996). Pure Appl. Chem..

[11] Bhattacharya A. (2004). Grafting: A versatile means to modify polymersTechniques, factors and applications. Prog. Polym. Sci..

[12] Yagci Y., Jockusch S., Turro N.J. (2010). Photoinitiated Polymerization: Advances, Challenges, and Opportunities. Macromolecules.

[13] Bowman C., Kloxin C.J. (2008). Toward an enhanced understanding and implementation of photopolymerization reactions. AIChE J..

[14] Priola A., Gozzelino G., Ferrero F., Malucelli G. (1993). Investigation on the structure-property relationships for films obtained from UV curable coatings. Prog. Org. Coat..

[15] Malucelli G., Gozzelino G., Bongiovanni R., Priola A. (1996). Photopolymerization of poly(tetramethylene ether) glycol diacrylates and properties of the obtained networks. Polymer.

[16] Sangermano M., Malucelli G., Morel F., Decker C., Priola A. (1999). Cationic photopolymerization of vinyl ether systems: Influence of the presence of hydrogen donor additives. Eur. Polym. J..

[17] Sangermano M., Bongiovanni R., Malucelli G., Priola A. (1999). Cationic photopolymerisation of divinylethers systems containing hydroxyvinylethers. Polym. Bull..

[18] Sangermano M., Spera S., Bongiovanni R., Priola A., Busetto C. (2000). NMR investigation of UV-cured vinyl ether networks. Macromol. Chem. Phys..

[19] Sangermano M., Malucelli G., Bongiovanni R., Priola A., Annby U., Rehnberg N. (2001). Cationic photopolymerization of polyfunctional 1-propenyl ether systems. Polym. Int..

[20] Sangermano M., Malucelli G., Bongiovanni R., Priola A., Annby U., Rehnberg N. (2002). Cationic photoinitiated copolymerization of 1-propenyl–vinyl ether systems. Eur. Polym. J..

[21] Sangermano M., Malucelli G., Bongiovanni R., Gozzelino G., Peditto F., Priola A. (2002). Coatings obtained through cationic UV curing of epoxide systems in the presence of epoxy functionalized polybutadiene. J. Mater. Sci..

[22] Bongiovanni R., Sangermano M., Malucelli G., Priola A., Leonardi A., Ameduri B., Pollicino A., Recca A. (2003). Fluorinated vinyl ethers as new surface agents in the photocationic polymerization of vinyl ether resins. J. Polym. Sci. Part A Polym. Chem..

[23] Sangermano M., Malucelli G., Bongiovanni R., Priola A. (2004). Photopolymerization of oxetane based systems. Eur. Polym. J..

[24] Sangermano M., Bongiovanni R., Malucelli G., Priola A., Olbrych J., Harden A., Rehnberg N. (2004). Synthesis and cationic photopolymerization of new silicon-containing oxetane monomers. J. Polym. Sci. Part A Polym. Chem..

[25] Sangermano M., Bongiovanni R., Malucelli G., Priola A., Thomas R., Medsker R., Kim Y., Kausch C. (2004). Synthesis and cationic photopolymerization of a new fluorinated oxetane monomer. Polymer.

[26] Crivello J.V., Lam J.H.W. (1977). Diaryliodonium Salts. A New Class of Photoinitiators for Cationic Polymerization. Macromolecules.

[27] Sangermano M., Roppolo I., Chiappone A. (2018). New Horizons in Cationic Photopolymerization. Polymers.

[28] Chatani S., Kloxin C.J., Bowman C. (2014). The power of light in polymer science: Photochemical processes to manipulate polymer formation, structure, and properties. Polym. Chem..

[29] Mariani A., Bidali S., Fiori S., Sangermano M., Malucelli G., Bongiovanni R., Priola A. (2004). UV-ignited frontal polymerization of an epoxy resin. J. Polym. Sci. Part A Polym. Chem..

[30] Kutal C., Grutsch P.A., Yang D.B. (1991). A novel strategy for photoinitiated anionic polymerization. Macromolecules.

[31] Abd-El-Aziz A.S., Antonietti M., Barner-Kowollik C., Binder W.H., Böker A., Boyer C., Buchmeiser M.R., Cheng S.Z.D., D’Agosto F., Floudas G. (2020). The Next 100 Years of Polymer Science. Macromol. Chem. Phys..

[32] Corrigan N., Yeow J., Judzewitsch P., Xu J., Boyer C. (2019). Seeing the Light: Advancing Materials Chemistry through Photopolymerization. Angew. Chem. Int. Ed..

[33] Galant O., Donmez H.B., Barner-Kowollik C., Diesendruck C.E. (2021). Flow Photochemistry for Single-Chain Polymer Nanoparticle Synthesis. Angew. Chem. Int. Ed..

[34] Dietlin C., Schweizer S., Xiao P., Zhang J., Morlet-Savary F., Graff B., Fouassier J.-P., Lalevée J. (2015). Photopolymerization upon LEDs: New photoinitiating systems and strategies. Polym. Chem..

[35] Sun K., Xiao P., Dumur F., Lalevée J. (2021). Organic dye-based photoinitiating systems for visible-light-induced photopolymerization. J. Appl. Polym. Sci..

[36] Houck H.A., Du Prez F.E., Barner-Kowollik C. (2017). Controlling thermal reactivity with different colors of light. Nat. Commun..

[37] Kuang X., Roach D.J., Wu J., Hamel C.M., Ding Z., Wang T., Dunn M.L., Qi H.J. (2019). Advances in 4D Printing: Materials and Applications. Adv. Funct. Mater..

[38] Spiegel C.A., Hippler M., Münchinger A., Bastmeyer M., Barner-Kowollik C., Wegener M., Blasco E. (2020). 4D Printing at the Microscale. Adv. Funct. Mater..

[39] Vitale A., Quaglio M., Marasso S.L., Chiodoni A., Cocuzza M., Bongiovanni R. (2013). Direct Photolithography of Perfluoropolyethers for Solvent-Resistant Microfluidics. Langmuir.

[40] Fantino E., Vitale A., Quaglio M., Cocuzza M., Pirri C.F., Bongiovanni R. (2015). Blue and UV combined photolithographic polymerization for the patterning of thick structures. Chem. Eng. J..

[41] Fantino E., Chiadò A., Quaglio M., Vaghi V., Cocuzza M., Marasso S.L., Potrich C., Lunelli L., Pederzolli C., Pirri C.F. (2020). Photofabrication of polymeric biomicrofluidics: New insights into material selection. Mater. Sci. Eng. C.

[42] Fantino E., Chiappone A., Roppolo I., Manfredi D., Bongiovanni R.M., Pirri C.F., Calignano F. (2016). 3D Printing of Conductive Complex Structures with In Situ Generation of Silver Nanoparticles. Adv. Mater..

[43] Wasser L., Vacche S.D., Karasu F., Müller L., Castellino M., Vitale A., Bongiovanni R., Leterrier Y. (2018). Bio-Inspired Fluorine-Free Self-Cleaning Polymer Coatings. Coatings.

[44] Kianfar P., Vitale A., Vacche S.D., Bongiovanni R. (2019). Photo-crosslinking of chitosan/poly(ethylene oxide) electrospun nanofibers. Carbohydr. Polym..

[45] Vitale A., Massaglia G., Chiodoni A., Bongiovanni R.M., Pirri C.F., Quaglio M. (2019). Tuning Porosity and Functionality of Electrospun Rubber Nanofiber Mats by Photo-Crosslinking. ACS Appl. Mater. Interfaces.

[46] Kianfar P., Vitale A., Vacche S.D., Bongiovanni R. (2021). Enhancing properties and water resistance of PEO-based electrospun nanofibrous membranes by photo-crosslinking. J. Mater. Sci..

[47] Garra P., Dietlin C., Morlet-Savary F., Dumur F., Gigmes D., Fouassier J.-P., Lalevée J. (2017). Photopolymerization processes of thick films and in shadow areas: A review for the access to composites. Polym. Chem..

[48] Mokbel H., Graff B., Dumur F., Lalevée J. (2020). NIR Sensitizer Operating under Long Wavelength (1064 nm) for Free Radical Photopolymerization Processes. Macromol. Rapid Commun..

[49] Yeow J., Chapman R., Gormley A.J., Boyer C. (2018). Up in the air: Oxygen tolerance in controlled/living radical polymerisation. Chem. Soc. Rev..

[50] Ligon S.C., Husár B., Wutzel H., Holman R., Liska R. (2013). Strategies to Reduce Oxygen Inhibition in Photoinduced Polymerization. Chem. Rev..

[51] Medellin A., Du W., Miao G., Zou J., Pei Z., Ma C. (2019). Vat Photopolymerization 3D Printing of Nanocomposites: A Literature Review. J. Micro Nano Manuf..

[52] Zhang Y., Xu Y., Simon-Masseron A., Lalevée J. (2021). Radical photoinitiation with LEDs and applications in the 3D printing of composites. Chem. Soc. Rev..

[53] Aparicio J.L., Elizalde M. (2015). Migration of Photoinitiators in Food Packaging: A Review. Packag. Technol. Sci..

[54] Chen H., Noirbent G., Sun K., Brunel D., Gigmes D., Morlet-Savary F., Zhang Y., Liu S., Xiao P., Dumur F. (2020). Photoinitiators derived from natural product scaffolds: Monochalcones in three-component photoinitiating systems and their applications in 3D printing. Polym. Chem..

[55] Molina-Gutiérrez S., Vacche S.D., Vitale A., Ladmiral V., Caillol S., Bongiovanni R., Lacroix-Desmazes P. (2020). Photoinduced Polymerization of Eugenol-Derived Methacrylates. Molecules.

[56] Vacche S.D., Vitale A., Bongiovanni R. (2019). Photocuring of Epoxidized Cardanol for Biobased Composites with Microfibrillated Cellulose. Molecules.

[57] Vacche S.D., Karunakaran V., Ronchetti S.M., Vitale A., Bongiovanni R. (2021). Nanocellulose from Unbleached Hemp Fibers as a Filler for Biobased Photocured Composites with Epoxidized Cardanol. J. Compos. Sci..

[58] Vitale A., Quaglio M., Chiodoni A., Bejtka K., Cocuzza M., Pirri C., Bongiovanni R. (2015). Oxygen-Inhibition Lithography for the Fabrication of Multipolymeric Structures. Adv. Mater..

[59] Cook C.J., Guymon C.A. (2014). Photo-Enforced Stratification of Polymeric Materials. U.S. Patent.

[60] Pollicino A., Siracusa V., Bongiovanni R., Priola A. (1995). X-ray photoelectron spectroscopic study of poly [4,4′-isopropylidenebis(1,4-phenyleneoxyethylene) diacrylate] photocured in the presence of a fluorine containing monomer. Macromol. Rapid Commun..

[61] Vitale A., Touzeau S., Sun F., Bongiovanni R. (2018). Compositional Gradients in Siloxane Copolymers by Photocontrolled Surface Segregation. Macromolecules.

